# Defining the genome structure of `Tongil' rice, an important cultivar in the Korean "Green Revolution"

**DOI:** 10.1186/s12284-014-0022-5

**Published:** 2014-09-14

**Authors:** Backki Kim, Dong-Gwan Kim, Gileung Lee, Jeonghwan Seo, Ik-Young Choi, Beom-Soon Choi, Tae-Jin Yang, Kwang Soo Kim, Joohyun Lee, Joong Hyoun Chin, Hee-Jong Koh

**Affiliations:** Department of Plant Science, Research Institute for Agriculture and Life Sciences, and Plant Genomics and Breeding Institute, Seoul National University, Seoul, 151-921 South Korea; National Instrumentation Center for Environmental Management (NICEM, Seoul National University, Seoul, 151-921 South Korea; PHYZEN Genome Institute, 501-1, Gwanak Century Tower, 1808 Nambusunhwan-ro, Gwanak-gu, Seoul, 151-836 South Korea; Department of Applied Bio Science, Konkuk University, Seoul, 143-701 South Korea; Plant Breeding, Genetics, and Biotechnology Division, International Rice Research Institute, DAPO 7777, Metro Manila, 1301 Philippines

**Keywords:** Tongil rice, Three-way cross, Next-generation sequencing, SEG map, Indica/japonica hybridization

## Abstract

**Background:**

Tongil (IR667-98-1-2) rice, developed in 1972, is a high-yield rice variety derived from a three-way cross between *indica* and *japonica* varieties. Tongil contributed to the self-sufficiency of staple food production in Korea during a period known as the `Korean Green Revolution'. We analyzed the nucleotide-level genome structure of Tongil rice and compared it to those of the parental varieties.

**Results:**

A total of 17.3 billion Illumina Hiseq reads, 47× genome coverage, were generated for Tongil rice. Three parental accessions of Tongil rice, two *indica* types and one *japonica* type, were also sequenced at approximately 30x genome coverage. A total of 2,149,991 SNPs were detected between Tongil and Nipponbare varieties. The average SNP frequency of Tongil was 5.77 per kb. Genome composition was determined based on SNP data by comparing Tongil with three parental genome sequences using the sliding window approach. Analyses revealed that 91.8% of the Tongil genome originated from the *indica* parents and 7.9% from the *japonica* parent. Copy numbers of SSR motifs, ORF gene distribution throughout the whole genome, gene ontology (GO) annotation, and some yield-related QTLs or gene locations were also comparatively analyzed between Tongil and parental varieties using sequence-based tools. Each genetic factor was transferred from the parents into Tongil rice in amounts that were in proportion to the whole genome composition.

**Conclusions:**

Tongil was derived from a three-way cross among two *indica* and one *japonica* varieties. Defining the genome structure of Tongil rice demonstrates that the Tongil genome is derived primarily from the *indica* genome with a small proportion of *japonica* genome introgression. Comparative gene distribution, SSR, GO, and yield-related gene analysis support the finding that the Tongil genome is primarily made up of the *indica* genome.

**Electronic supplementary material:**

The online version of this article (doi:10.1186/s12284-014-0022-5) contains supplementary material, which is available to authorized users.

## Background

Rice (*Oryza sativa* L.) is a staple food for more than half of the world's population, providing about 19 percent of the world's and 29 percent of Asia's caloric supply (IRRI [2009]). Although demands on the nutritional and industrial functionality of rice are increasing, especially to improve human health and quality of life, improving the yield potential of rice is still a major challenge for rice breeders, who must address the rapid growth of the world population along with dramatic reductions in the amount of cultivated land (Khush [1999]), as well as environmental challenges (Nelson, International Food Policy Research Institute [2009]). Asian varieties of cultivated rice include two major subspecies, *O. sativa indica* and *O. s. japonica,* which are differentiated based on morphological and physiological characteristics and geographical distribution (Morishima and Oka [1981]; Sano and Morishima [1992]). *O. s. indica* cultivars have higher genetic diversity (Lu et al. [2002]), a broader cultivation range, and stronger resistance to prominent diseases and insect pests compared to *O. s. japonica* cultivars (Chung and Heu [1991]). Inter-subspecific hybridization between *indica* and *japonica* rice cultivars may enrich allelic variation and facilitate hybrid vigor by creating new genetic recombinations (Cheng et al. [2007]). In spite of these advantages, the introduction of desirable *indica* traits into the *japonica* variety has not been successful due to reproductive barriers and the incorporation of undesirable characteristics, such as low eating quality for people who prefer the taste of *japonica* rice (Chung and Heu [1991]).

**Figure 1:**
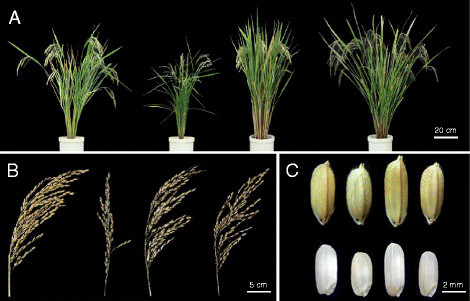
**Morphological comparison of Tongil and parental lines.** From left to right: Tongil, Yukara, IR8, and TN1. **(A)** The plant architecture of Tongil, its *japonica* parent (Yukara), and its *indica* parents (IR8 and TN1). **(B)** The panicle phenotype of Tongil and its parents. **(C)** The grain shapes and brown rice shapes of Tongil and its parents. Scale bars are included in each panel.

Tongil rice (IR667-98-1-2) is the first semi-dwarf variety obtained by a three-way cross of *indica*/*japonica* varieties as part of a collaborative research project between the International Rice Research Institute (IRRI) and the government of South Korea (Figure 1). The development of Tongil rice resulted in a significant yield increase from 4 to 5 t ha^-1^, corresponding to a 30% yield increase relative to the leading *japonica* varieties grown in Korea (Chung and Heu [1980]). After the introduction of Tongil rice in 1972, Korean rice production significantly increased and the South Korean government announced the achievement of agricultural self-sufficiency (the so-called `Green Revolution') in 1977. However, the genome characterization and structure of Tongil rice have never been analyzed.

Rice is a useful model crop for studying genome structure due to its relatively small genome. Furthermore, its genetic and physical data have been extensively analyzed by the International Rice Genome Sequencing Project (IRGSP) (International Rice Genome Sequencing P [2005]). The recent improvement of next-generation sequencing (NGS) technology has enabled high-throughput genotyping and elucidation of genome structures of various rice cultivars (Huang et al. [2009]; Huang et al. [2012]). Most sequence-based rice genome analyses are based on DNA polymorphisms, single nucleotide polymorphisms (SNPs) and insertion-deletions (InDels). SNP detection is the first step for comparing DNA variation and is an effective tool to elucidate genome structure and composition (Feltus et al. [2004]; McNally et al. [2009]; Shen et al. [2010]; Chen et al. [2014]).

In this study, we sequenced the whole genomes of Tongil rice (*Oryza sativa* L.) and its parental varieties to analyze the genome structure of Tongil in detail and to identify regions of the *indica* and *japonica* parental genomes that introgressed in the Tongil genome. In addition, we analyzed previously reported yield-related genes (*Gn1a*, *Ghd7*, *sd1*, *GS3* and *qSW5*), SSRs, GO annotation, and other genetic characteristics of the Tongil genome.

## Results

### Genome structure of Tongil

**Figure 2:**
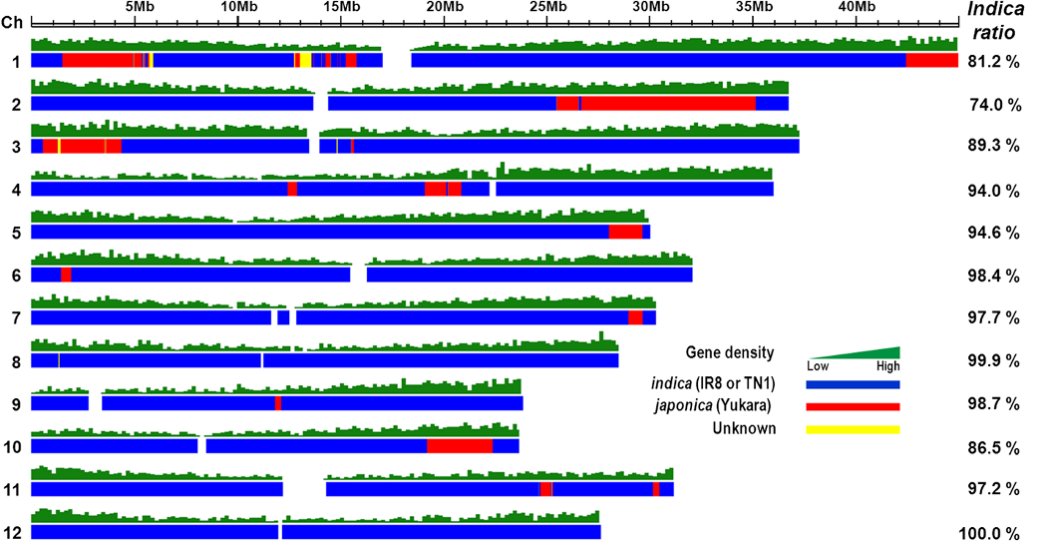
***Indica*** **/** ***japonica*** **genome organization on the 12 chromosomes of Tongil.** Blue indicates the *indica* genome (TN1 and IR8); red indicates the *japonica* genome (Yukara); and yellow indicates a region from an unknown genome. The percentages describe the proportion of *indica* contribution on each chromosome.

The whole genomes of Tongil and its three parental varieties, Yukara, IR8, and TN1 (Taichung Native 1), were sequenced on the Illumina-GAII platform. A large number of short reads were mapped onto the reference Nipponbare genome and then assembled into a consensus sequence. A total of 199,543,820 reads of the Tongil genome, corresponding to 17,339,883,560 bp (17.3 Gb), were generated, representing a 47-fold sequence depth and covering 88.8% of the Nipponbare pseudomolecules (Table 1 and Additional file 1: Table S1). We detected a total of 2,149,991 SNPs between Tongil and Nipponbare sequences (Additional file 2: Table S2). The two *indica* parents of Tongil, IR8 and TN1, had 6.22 and 6.04 SNPs per kb, respectively, whereas the *japonica* parent of Tongil, Yukara, had only 0.49 SNP per kb (Additional file 2: Table S2). Using the SNP data sets from Tongil and its parents, we defined the genomic origins of regions of the Tongil genome by SNP calling (Additional file 3: Figure S1; Additional file 4: Table S3; see also the SNP calling section in the Materials and Methods), and then performed a SEG-Map analysis (Zhao et al. [2010b]) of Tongil (Figure 2). The whole genome of Tongil consisted of an average contribution of 91.8% from *indica*, 7.9% from *japonica*, and 0.3% unknown (i.e., not defined as *indica* or *japonica* regions) (Figure 2 and Table 2). The contribution of *indica* to the Tongil genome varied across chromosomes, from 74% (Chr. 2) to 100% (Chr. 12). A relatively high proportion of the *japonica* genome was found on chromosomes 1, 2, and 3, whereas the *japonica* sequences were barely detectable on chromosomes 8 and 12. In addition, there were no differences in gene density between the *indica-* and *japonica*-derived genome regions of Tongil (Figure 2 and Table 2).

**Table 1:** **General sequencing statistics for Tongil and its parental genomes**

Variety	Number of reads	Total read length (bp)	Mapped read length (bp)	Sequencing depth (×)	Coverage^a)^(%)	SNP frequency (SNPs/kb)
Tongil	199,543,820	17,339,883,560	330,933,489	47	88.8	5.77
Yukara	114,615,268	12,429,060,750	345,058,384	34	92.6	0.49
IR8	109,304,614	11,790,909,253	327,065,806	32	87.7	6.22
TN1	105,708,026	11,299,286,038	326,132,058	30	87.5	6.04

^a)^Coverage to Nipponbare genome sequence.

Sequencing and mapping against the Nipponbare reference genome.

**Table 2:** **Determination of the** ***indica*** **/** ***japonica*** **genome origin of Tongil, based on a window size of 9**

Chromosome	Pseudomolecule	***Indica***region (bp)	Ratio (%)	***Japonica***region (bp)	Ratio (%)	Unknown region (bp)	Ratio (%)
1	45,038,604	36,563,905	81.2	7,596,808	16.9	877,891	2.0
2	36,792,247	27,235,850	74.0	9,544,379	25.9	12,018	0.0
3	37,312,367	33,336,733	89.3	3,748,667	10.1	226,967	0.6
4	36,060,865	33,898,364	94.0	2,150,911	6.0	11,590	0.0
5	30,073,438	28,436,341	94.6	1,637,097	5.4	-	-
6	32,124,789	31,619,689	98.4	499,676	1.6	5,424	0.0
7	30,357,780	29,667,148	97.7	690,632	2.3	-	-
8	28,530,027	28,487,631	99.9	333	-	42,063	0.2
9	23,895,721	23,592,877	98.7	302,844	1.3	-	-
10	23,703,430	20,504,662	86.5	3,198,768	13.5	-	-
11	31,219,694	30,345,040	97.2	846,802	2.7	27,852	0.1
12	27,679,166	27,679,166	100.0	-	-	-	-
Total	382,788,128	351,367,406	91.8	30,216,917	7.9	1,203,805	0.3

### Gene distribution and gene ontology analysis of Tongil

We analyzed the gene content of Tongil to understand the relationship between the composition of the genome and genes (open reading frames: ORFs), and also to elucidate the distribution of *indica-* and *japonica-* originated genes (alleles) within the Tongil genome. The gene distribution ratio according to *indica* or *japonica* genome composition was similar to the genome distribution ratio (Table 2 and Additional file 5: Table S4). The origins of genes from the *indica* and *japonica* parents were 88.3% and 11.4%, respectively, suggesting that the average gene composition was similar to the genome composition ratio of Tongil, although the distribution of parental origin varied across chromosomes. We performed gene ontology (GO) analysis of the Tongil genome according to three categories to identify biological patterns using a list of genes derived from *indica*, *japonica*, and unknown genomes: cellular components, molecular functions, and biological processes (Additional file 6: Figure S2; Additional file 7: Figure S3; Additional file 8: Figure S4). The results of GO analysis revealed that the average contribution of the *indica* or *japonica* genome to each GO category was almost identical to the gene and genome distribution ratios. *O. s. indica* and *O. s. japonica* contributed 86.8% and 12.7% of the cellular components, 87.4% and 12.2% of the molecular functions, and 87.3% and 12.2% of the biological processes, respectively, to the Tongil genome. However, in the `molecular functions' category, all 17 genes related to channel regulator activity were derived from *indica* regions, whereas all adhesion-related genes in the biological processes category were derived solely from *japonica* regions.

### Simple sequence repeats (SSRs) in the Tongil genome

A total of 177 distinctive motif families were annotated on the Tongil genome (Additional file 9: Figure S5; Additional file 10: Figure S6). Di-nucleotide repeats were predominant among the classified repeats, and AT/TA repeats were the most abundant motifs in both *indica-* (29.09%) and *japonica-* derived (21.8%) regions within the Tongil genome. The next most abundant motif relative to AT/TA was CT/GA, and CGC was the most abundant motif among tri-nucleotide repeats. The di-, tri-, and tetra-nucleotide repeat patterns were different from that of the reference Nipponbare genome (McCouch et al. [2002]; Zhou et al. [2005]), and also differed from that of wheat (Weng et al. [2005]). A total of 90.1% of SSR motifs in the Tongil genome were from *indica*, 9.6% were from *japonica*, and 0.3% were from an unknown genome (Additional file 10: Figure S6).

### Distribution of yield-related genes in the Tongil genome

One of the most important aims of this study was to explore which regions of the *indica* and *japonica* parental genomes have introgressed into the Tongil variety to provide its high-yield potential. Tongil is morphologically characterized by short plant height, lodging resistance, open plant architecture, medium-long erect leaves, thick leaf sheaths and culms, relatively long panicles, and easily shattered grain (Chung and Heu [1980]) (Figure 1). Although these phenotypic characteristics affect Tongil's high-yield potential, to date we have no molecular genetic evidence regarding the nature of these traits, with the exception of semi-dwarf gene 1 (*sd1*) (Chung and Heu [1980]). Therefore, we analyzed several well-characterized genes associated with high yield potential in the Tongil genome: *sd1* (Nagano et al. [2005]; Sasaki et al. [2002]; Monna et al. [2002]), *Ghd7* (Liu et al. [2013]; Xue et al. [2008]), *Gn1a* (Ashikari et al. [2005]; Miura et al. [2010]), *qSW5* (Yan et al. [2011]; Shomura et al. [2008]), *GS3* (Takano-Kai et al. [2009]; Fan et al. [2006]), and *GW2* (Li et al. [2010]; Song et al. [2007]).

#### sd(semi-dwarf stature)

**Figure 3:**
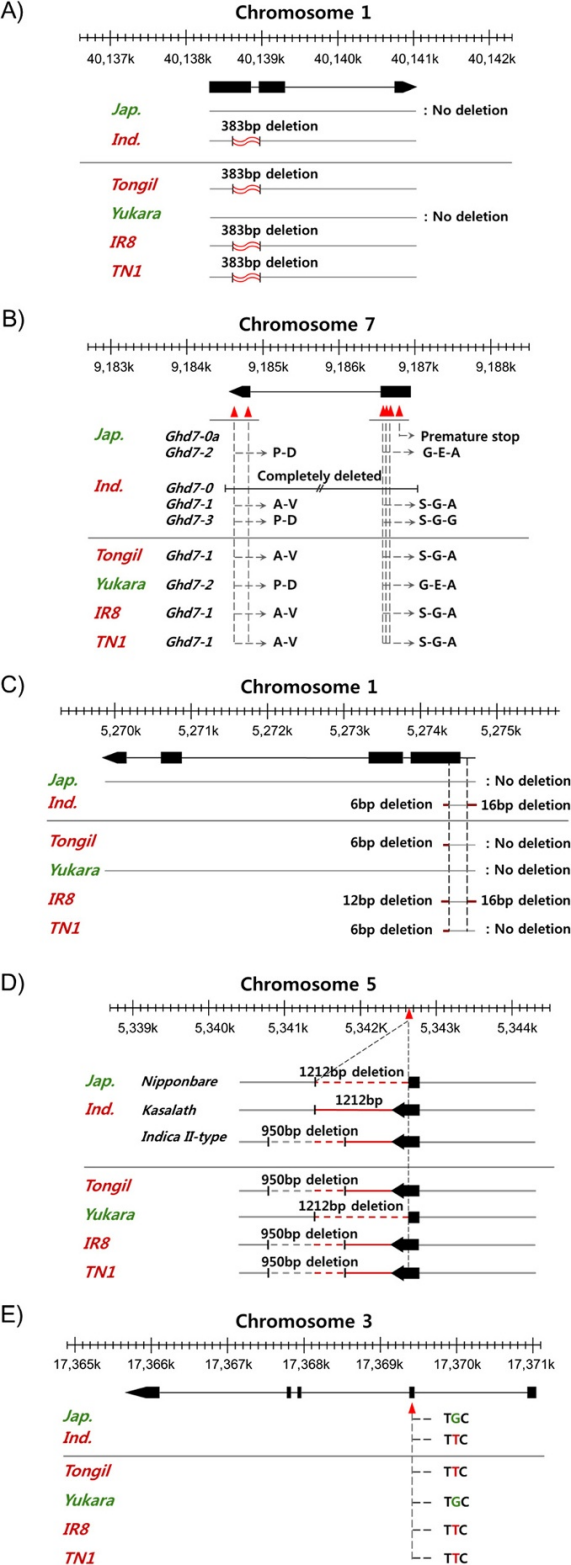
***Indica*** **/** ***japonica*** **region comparisons of high yield-related alleles or QTLs. A)** *sd1*, **B)** *Ghd7*, **C)** *Gn1a*, **D)** *qSW5*, and **E)** *GS3*. Black arrows and box regions represent exons. Vertical, dashed lines refer to the same position in the genome or gene region.

Semi-dwarf stature is one of the main genetic contributors to the success of the Green Revolution. The introduction of semi-dwarf genes increased yield by conferring lodging resistance, which enabled greater input of nitrogen fertilizer. Tongil was the first variety into which the *sd1* allele was introduced in South Korea. Analysis of the *sd1* gene, which encodes GA20ox-2 in Tongil and its parents, revealed that Tongil received its *sd1* from an *indica* parent, IR8 or TN1; this allele contained a 383-bp deletion resulting in a frame-shift to form a stop codon (Figure 3A). We also confirmed other *sd1* alleles derived from the native semi-dwarf rice variety Jikkoku (G281T) and the γ-ray-induced varieties Reimei (C1045G) and Calose76 (C796T) (Monna et al. [2002]; Sasaki et al. [2002]). However, Yukara, the *japonica* parent of Tongil, did not have any *sd1* alleles.

#### Ghd(grain number, plant height, and heading date)

A gene encoding a CCT domain protein, *Ghd7*, is an important regulator of potential yield, plant height, and heading date in rice. Plant height and panicle size are increased under long-day conditions by the delay in heading date resulting from increased *Ghd7* expression. *Ghd7* has five natural variant haplotypes (Xue et al. [2008]). Among these, Tongil possesses the *Ghd7-1* allele (A-G-S-V-A) derived from *indica* parent IR8 or TN1 (Figure 3B), which is considered to be the original, fully-functional wild-type allele; plants with this allele are relatively tall, late heading, have large panicles, and are widely grown. By contrast, the *japonica* parent of Tongil, Yukara, has the *Ghd7-2* allele (A-E-G-D-P), which is weaker than *Ghd7-1* and is found in temperate *japonica* varieties.

#### Gn1a(Grain number on chromosome 1)

*Gn1a* is one of the most effective QTLs for increasing grain number. It is predicted to encode a cytokinin oxidase/dehydrogenase (OsCKX2). Habataki, an *indica* rice variety, has a 16-bp deletion in the 5' UTR, a 6-bp deletion in the first exon, and three amino acid substitutions in the first and fourth exons of this gene. In addition, an 11-bp deletion in the third exon has been detected in the high-yielding rice variety 5150 (Ashikari et al. [2005]). Comparisons of DNA sequences between Tongil and parent varieties revealed that the Tongil sequence was identical to the TN1 allele, which had only a 6-bp deletion in the first exon and no 16 bp deletion in the 5' UTR, as in Habataki. On the other hand, IR8 contained a 16-bp deletion in the 5' UTR and a 12-bp deletion in the first exon, distinct from the pattern in the TN1 allele. We could not identify any variation in Yukara, which has the same allele sequence as Nipponbare (Figure 3C).

#### qSW5(QTL for seed width on chromosome 5)

*qSW5* is responsible for seed width; the product of this gene controls cell number in the outer glume of the rice flower. The gene product increases seed width and seed weight by enlarging sink size. The Nipponbare-type allele, which contains a 1,212-bp deletion, is a loss-of-function allele relative to the Kasalath-type allele (Shomura et al. [2008]). In addition, the *indica* II-type allele has a 950-bp deletion relative to the Kasalath allele (Yan et al. [2011]). Comparisons of the *qSW5* alleles between Tongil and parental varieties revealed that Tongil, IR8, and TN1 have the *indica* II-type allele, whereas Yukara has the Nipponbare allele (Figure 3D).

#### GS3(Grain length and weight; grain size 3)

*GS3*, which encodes a PEPB-like domain protein, was cloned from a QTL for grain length and weight on chromosome 3 in rice. A C-to-A substitution in the second exon of the *GS3* gene is strongly associated with grain length and width: the A-allele confers significantly longer and thinner grains than the C-allele (Takano-Kai et al. [2009]; Fan et al. [2006]). Tongil possesses an A-allele originating from an *indica* parent, IR8 or TN1, whereas the *japonica* parent, Yukara has the C-allele (Figure 3E). In the case of another gene that controls grain width, *GW2*, there were no SNPs detected among any of the strains we sequenced or Nipponbare, indicating that GW2 is a highly conserved gene in rice and even in *Zea mays* (Li et al. [2010]; Song et al. [2007]).

## Discussion

In this study, we used high-depth NGS analysis to demonstrate that the Tongil genome is composed of 91.8% *indica*, 7.9% *japonica*, and 0.3% unknown genome. The amounts and types of genes and SSRs in the Tongil genome were very similar to its genomic composition with respect to *indica* or *japonica* origin. This deviation from the expectation that about one-fourth of the Tongil genome originated from the *japonica* parent is likely due to the results of selection during the breeding process and/or to segregation distortion in favor of the *indica* genome because *indica*-type alleles and plants are favored among hybrid progenies from *indica*/*japonica* crosses (Harushima et al. [1996]; Lin et al. [1992]).

Tongil rice is highly successful in terms of grain yield in South Korea, although Korean climatic environments are not favorable to the cultivation of typical *indica* varieties (Chung and Heu [1991]). This may be attributable to its heightened adaptability compared to most *indica* varieties, perhaps due to the partial incorporation of the *japonica* parental genome.

From an agronomic viewpoint, rice yield is determined by the integration of four yield components: number of panicles per unit area, number of grains per panicle, filled grain ratio, and grain weight. Tongil is a heavy-panicle variety with more grains per panicle than its parental varieties (Chung and Heu [1991]). We manually sequenced the yield-related genes *sd1*, *Gn1a*, *Ghd7*, *GS3*, *qSW5*, and *GW2* to determine which alleles came from the *indica* and *japonica* parents (Figure 3; Additional file 11: Table S5). The *sd1*, *Gn1a*, and *Ghd7* alleles of Tongil originated from the *indica* parents, as did the *GS3*, *qSW5*, and *GW2* alleles of Tongil, all of which are involved in determining seed size. Although Tongil and its *indica* parents share the same allele for these three genes, the seed shape of Tongil is closer to TN1 than that of IR8 (Figure 1). Thus, another gene or epistatic interaction may be involved in determining seed shape in the Tongil cultivar (Yan et al. [2011]). In fact, all of the alleles identified in Tongil were the same as those in TN1, one of the *indica* parents. Therefore, it is unlikely that a greater understanding of the high yield potential of Tongil could be achieved by analyzing these six yield-related genes. Complex genetic systems, including unknown genes and epistatic interactions, should be investigated in future studies.

Since the initial success of Tongil rice in Korea, numerous Tongil-type varieties of similar parentage or that were bred using Tongil rice as one of the parents have been developed to address future needs for food security. We predict that genomic information, including the SNP data provided in this study, will facilitate the efficient breeding of these and other Tongil-type varieties.

## Conclusions

We determined the genome structure of Tongil rice, a successful cultivar derived from *indica* × *japonica* hybridization in Korea. Analyses of genome composition and genetic factors of Tongil rice demonstrate that the Tongil genome is derived mostly from the *indica* genome, with a small portion of *japonica* genome introgression. The approach used in this study to determine the parental origins of specific genome segments is applicable to the genomic dissection of agricultural breeding lines or varieties of diverse parental origins.

## Methods

### Plant materials

Plant lines subjected to whole-genome resequencing in the present study included Tongil (SNU accession no. 260697) and its parental lines: Yukara, an early maturing temperate *japonica* cultivar (RDA-Genebank Information Center accession no. IT004665); Taichung native 1 (TN1), the first semi-dwarf *indica* variety with high adaptability (RDA-Genebank Information Center accession no. IT004120); and IR8, an improved high-yielding semi-dwarf variety developed at the International Rice Research Institute (IRRI, IRTP 195). The Tongil variety was developed through a three-way cross, IR8//Yukara/TN1. With generation advancement after the cross, the most promising line, IR667-98-1-2, was selected and released to farmers in Korea under the name `Tongil' (Chung and Heu [1991]).

### Whole-genome DNA sequencing

Four rice varieties were sequenced: Tongil and its parental varieties, Yukara, IR8, and TN1. Whole-genome shotgun sequencing of the four rice genomes was performed using the Illumina/Solexa GAII system. DNA sequencing, including construction of shotgun DNA libraries, was performed according to the methods recommended by the manufacturer (Illumina, San Diego, CA, USA). Briefly, whole-genome DNA shotgun paired-end sequencing libraries were generated by fragmentation of DNA into 500-bp segments using a Covaris DNA shearing machine (Covaris, CA, US), followed by ligation of paired-end adapters ligation of 53 and 68 bp for sequencing on the FlowCell, size selection of the adapter-ligated fragments within the desired size range (500-600 bp), and PCR enrichment using complete primer constructs required for binding and clustering on the FlowCell. Illumina GAII sequencing was performed by identifying the emission color of single-base extensions on the FlowCell.

### DNA variation

Illumina whole-genome shotgun 100-bp paired-end DNA sequencing data were filtered to obtain high-quality sequence data and to map reads to the Nipponbare reference genome sequence, which as downloaded from NCBI. Briefly, high-quality sequence with at least QC20-justified phred quality score was mapped to the reference Nipponbare sequence using CLC NGS Cell software (http://www.clcbio.com). The DNA sequence variation DB was converted to text format, including DNA variation based on the reference position, for the analysis of genome structure.

### SNP calling - probabilities

Genotype calling to identify regions originating from the *japonica* and *indica* genomes was performed using the sliding-window approach suggested by Huang et al. (Huang et al. [2009]). In each window, the proportion of SNPs originating from each parent was examined for genotype calling. Huang et al. determined optimum window size by calculating the probability of finding a specific number of *japonica* SNPs in a window based on SNP error rates. Recent improvements in sequencing technology, however, resulted in fewer errors in SNP identification. Thus, the method suggested by Huang et al. ([2009]) was not directly applicable in this study. Even with a window size of 2, for example, calling accuracy could reach 99.99%. Instead of calculating this probability, the optimum window size was determined iteratively by comparing the portion of *japonica* SNPs (*O*) and the portion of the genome originating from *japonica* (*P*). Tongil was resequenced to obtain SNPs originating from its parents and to calculate the percentage of *japonica* SNPs in each chromosome. SEG-Map software (Huang et al. [2009]) was also used for genotype calling on each chromosome. Because the optimum window size was unknown, a range of window sizes from 1 to 199 was used. Then, the Nash-Sutcliffe efficiency (*E*) between *O* and *P* was calculated as follows:

1 $$E=1-\frac{\sum_{i=1}^{n}{\left(O_{i}-P_{i}\right)}^{2}}{\sum_{i=1}^{n}{\left(O_{i}-O_{m}\right)}^{2}}$$

Here, an individual chromosome is denoted by *i*. The average percentage of *japonica* SNPs on each chromosome is denoted by O_m_. The optimal window size was defined as that with a maximum value of *E*; values of *E* ranged from -29 to 0.963. This maximum value of E occurred with a window size of 9. The percentage of *indica* SNPs was at its second highest (0.966) with a window size of 9. At a window size of 10, the *E* value dropped rapidly for *japonica* SNPs (0.037) and *indica* SNPs (-0.018). Thus, a window size of 9 was selected as the optimum for data analysis (Additional file 7: Figure S3).

### Parental genome composition of Tongil

We compared DNA variation between the parental and Tongil genomes. Genomic regions originating from the *japonica* (Yukara) and *indica* (TN1 or IR8) parents were identified by comparing the Tongil genome sequence to parental sequences. Estimated *indica* and *japonica* regions in the Tongil genome sequence were calculated based on the methods of Zhao et al. (Zhao et al. [2010a]).

### Gene ontology and classification

Annotated Nipponbare reference genes were classified based on parental origin in the Tongil genome and assigned to the three main GO-term categories (cellular component, molecular function, and biological process) using BLAST2GO software (www.blast2go.com) (Conesa et al. [2005]).

### Simple sequence repeats (SSRs)

SSR loci were searched using SSR search software (Initiative [2000]) and classified with respect to their parental origin.

## Authors' contributions

BK and HK conceived of the study and participated in its design. IC and BC performed bioinformatic analysis and data processing. BK and JL collected samples and phenotype data. DK, BK, GL, and JS analyzed the data and helped to draft the manuscript. TY, KK, DK, and JC helped to revise the manuscript. All authors read and approved the final manuscript.

## Additional files

## Electronic supplementary material

Additional file 1: Table S1.: Mapping coverage of Tongil rice and its three parents. (DOCX 21 KB)

Additional file 2: Table S2.: SNPs and SNP frequency of Tongil and its three parents. (DOCX 21 KB)

Additional file 3: Figure S1.: Determination of window size followed by *E*-value calculation. The x-axis is the window size and the y-axis is the calculated *E*-value. (DOCX 24 KB)

Additional file 4: Table S3.: Genome region definition by the presence (O) or absence (X) of SNPs. (DOCX 19 KB)

Additional file 5:Table S4.: Gene distribution of Tongil. (DOCX 20 KB)

Additional file 6: Figure S2.: GO analysis according to the cellular components category of Tongil genes corresponding to *indica*/*japonica* sequences. (DOCX 22 KB)

Additional file 7: Figure S3.: GO analysis according to the molecular functions category of Tongil genes corresponding to *indica*/*japonica* sequences. (DOCX 22 KB)

Additional file 8: Figure S4.: GO analysis according to the biological processes category of Tongil genes corresponding to *indica*/*japonica* sequences. (DOCX 22 KB)

Additional file 9: Figure S5.: Copy number of SSR motif families in Tongil. (DOCX 1 MB)

Additional file 10: Figure S6.: List of SSR motif families in Tongil. (XLSX 1 MB)

Additional file 11: Table S5.: Comparison of alleles of yield-related genes in Tongil and its parents. (DOCX 20 KB)

Below are the links to the authors’ original submitted files for images.

Authors’ original file for figure 1

Authors’ original file for figure 2

Authors’ original file for figure 3

## Abbreviations

SNP: Single nucleotide polymorphism
NGS: Next generation sequencing
IRGSP: International rice genome sequencing project
SSR: Simple sequence repeat
GO: Gene ontology
SEG-map: Sequencing enabled genotyping for mapping recombination populations
