# Supplementary material for: Defining the genome structure of `Tongil' rice, an important cultivar in the Korean "Green Revolution"
Source: Rice (N Y). 2014 Sep 14;7:22. doi: 10.1186/s12284-014-0022-5 (PMC4883996; doi:10.1186/s12284-014-0022-5)
Supplement: Supplementary file 1 — Additional file 1: Table S1.: Mapping coverage of Tongil rice and its three parents. (DOCX 21 KB) [file 12284_2014_22_MOESM1_ESM.docx]

Table S1 Mapping coverage of Tongil rice and its three parents

|  | Reference |  | Tongil | |  | Yukara | |  | IR8 | |  | TN1 | |
| --- | --- | --- | --- | --- | --- | --- | --- | --- | --- | --- | --- | --- | --- |
| Chromosome | Pseudomolecule |  | Aligned  Length (bp) | Coverage (%) |  | Aligned  Length (bp) | Coverage (%) |  | Aligned  Length (bp) | Coverage (%) |  | Aligned  Length (bp) | Coverage (%) |
| 1 | 45,038,604 |  | 38,574,603 | 89.2 |  | 39,521,561 | 91.4 |  | 37,917,584 | 87.7 |  | 37,633,042 | 87.0 |
| 2 | 36,792,247 |  | 32,784,930 | 91.2 |  | 33,736,987 | 93.9 |  | 32,281,381 | 89.8 |  | 32,249,409 | 89.8 |
| 3 | 37,312,367 |  | 33,412,277 | 91.9 |  | 34,084,555 | 93.7 |  | 33,168,624 | 91.2 |  | 33,108,547 | 91.0 |
| 4 | 36,060,865 |  | 31,735,280 | 89.3 |  | 33,210,463 | 93.5 |  | 31,468,452 | 88.6 |  | 31,193,791 | 87.8 |
| 5 | 30,073,438 |  | 27,991,645 | 93.5 |  | 28,624,984 | 95.6 |  | 27,856,337 | 93.0 |  | 27,770,415 | 92.7 |
| 6 | 32,124,789 |  | 27,784,237 | 89.0 |  | 28,323,987 | 90.7 |  | 27,516,550 | 88.1 |  | 27,400,223 | 87.7 |
| 7 | 30,357,780 |  | 26,328,583 | 88.6 |  | 27,297,454 | 91.8 |  | 25,826,093 | 86.9 |  | 25,896,533 | 87.1 |
| 8 | 28,530,027 |  | 25,808,735 | 90.7 |  | 27,150,402 | 95.5 |  | 25,591,577 | 90.0 |  | 25,618,079 | 90.1 |
| 9 | 23,895,721 |  | 19,770,440 | 85.9 |  | 21,074,657 | 91.6 |  | 19,774,924 | 86.0 |  | 19,460,581 | 84.6 |
| 10 | 23,703,430 |  | 20,191,910 | 88.0 |  | 21,233,194 | 92.5 |  | 19,872,700 | 86.6 |  | 19,739,332 | 86.0 |
| 11 | 31,219,694 |  | 22,752,159 | 78.7 |  | 24,726,750 | 85.5 |  | 22,345,311 | 77.3 |  | 22,512,524 | 77.8 |
| 12 | 27,679,166 |  | 23,798,690 | 86.4 |  | 26,073,390 | 94.7 |  | 23,446,273 | 85.2 |  | 23,549,582 | 85.5 |
| Total or Ave. | 382,788,128 |  | 330,933,489 | 88.8 |  | 345,058,384 | 92.6 |  | 327,065,806 | 87.7 |  | 326,132,058 | 87.5 |
