# Supplementary material for: Defining the genome structure of `Tongil' rice, an important cultivar in the Korean "Green Revolution"
Source: Rice (N Y). 2014 Sep 14;7:22. doi: 10.1186/s12284-014-0022-5 (PMC4883996; doi:10.1186/s12284-014-0022-5)
Supplement: Supplementary file 2 — Additional file 2: Table S2.: SNPs and SNP frequency of Tongil and its three parents. (DOCX 21 KB) [file 12284_2014_22_MOESM2_ESM.docx]

Table S2 SNPs and SNP frequency of Tongil and its three parents

|  | Reference |  | Tongil | |  | Yukara | |  | IR8 | |  | TN1 | |
| --- | --- | --- | --- | --- | --- | --- | --- | --- | --- | --- | --- | --- | --- |
| Chromosome | Pseudomolecule |  | Number  of SNP | SNP Frequency (SNPs/Kbp) |  | Number  of SNP | SNP Frequency (SNPs/Kbp) |  | Number  of SNP | SNP Frequency (SNPs/Kbp) |  | Number  of SNP | SNP Frequency (SNPs/Kbp) |
| 1 | 45,038,604 |  | 222,438 | 5.14 |  | 19,609 | 0.45 |  | 267,113 | 6.18 |  | 262,349 | 6.07 |
| 2 | 36,792,247 |  | 159,177 | 4.43 |  | 7,129 | 0.20 |  | 225,762 | 6.28 |  | 209,537 | 5.83 |
| 3 | 37,312,367 |  | 183,617 | 5.05 |  | 6,785 | 0.19 |  | 205,566 | 5.65 |  | 192,339 | 5.29 |
| 4 | 36,060,865 |  | 177,722 | 5.00 |  | 24,186 | 0.68 |  | 190,976 | 5.38 |  | 182,173 | 5.13 |
| 5 | 30,073,438 |  | 132,916 | 4.44 |  | 3,276 | 0.11 |  | 141,789 | 4.73 |  | 145,396 | 4.85 |
| 6 | 32,124,789 |  | 184,344 | 5.90 |  | 23,105 | 0.74 |  | 187,503 | 6.00 |  | 184,005 | 5.89 |
| 7 | 30,357,780 |  | 198,051 | 6.66 |  | 21,460 | 0.72 |  | 205,384 | 6.91 |  | 191,745 | 6.45 |
| 8 | 28,530,027 |  | 162,946 | 5.73 |  | 15,459 | 0.54 |  | 163,856 | 5.76 |  | 162,534 | 5.71 |
| 9 | 23,895,721 |  | 154,565 | 6.72 |  | 11,273 | 0.49 |  | 143,014 | 6.22 |  | 150,864 | 6.56 |
| 10 | 23,703,430 |  | 146,848 | 6.40 |  | 6,749 | 0.29 |  | 163,379 | 7.12 |  | 165,561 | 7.22 |
| 11 | 31,219,694 |  | 226,457 | 7.83 |  | 28,203 | 0.98 |  | 227,571 | 7.87 |  | 216,814 | 7.50 |
| 12 | 27,679,166 |  | 200,910 | 7.30 |  | 17,131 | 0.62 |  | 198,404 | 7.21 |  | 190,243 | 6.91 |
| Total or Ave. | 382,788,128 |  | 2,149,991 | 5.77 |  | 184,365 | 0.49 |  | 2,320,317 | 6.22 |  | 2,253,560 | 6.04 |
