# Supplementary material for: Defining the genome structure of `Tongil' rice, an important cultivar in the Korean "Green Revolution"
Source: Rice (N Y). 2014 Sep 14;7:22. doi: 10.1186/s12284-014-0022-5 (PMC4883996; doi:10.1186/s12284-014-0022-5)
Supplement: Supplementary file 3 — Additional file 3: Figure S1.: Determination of window size followed by E-value calculation. The x-axis is the window size and the y-axis is the calculated E-value. (DOCX 24 KB) [file 12284_2014_22_MOESM3_ESM.docx]

Figure S1 Determination of window size followed by *E*-value calculation
