# Supplementary material for: Defining the genome structure of `Tongil' rice, an important cultivar in the Korean "Green Revolution"
Source: Rice (N Y). 2014 Sep 14;7:22. doi: 10.1186/s12284-014-0022-5 (PMC4883996; doi:10.1186/s12284-014-0022-5)
Supplement: Supplementary file 4 — Additional file 4: Table S3.: Genome region definition by the presence (O) or absence (X) of SNPs. (DOCX 19 KB) [file 12284_2014_22_MOESM4_ESM.docx]

Table S3 Genome region definition by the presence (Ｏ) or absence (Ｘ) of SNPs

| Genome origin from / Define as | Presence of SNP or not | | |
| --- | --- | --- | --- |
|  | Yukara | IR8 | TN1 |
| IR8 / *indica* | Ｏ | Ｘ | Ｏ |
| TN1 / *indica* | Ｏ | Ｏ | Ｘ |
| IR8 and TN1 / *indica* | Ｏ | Ｘ | Ｘ |
| Yukara / *japonica* | Ｘ | Ｏ | Ｏ |
