# Supplementary material for: Defining the genome structure of `Tongil' rice, an important cultivar in the Korean "Green Revolution"
Source: Rice (N Y). 2014 Sep 14;7:22. doi: 10.1186/s12284-014-0022-5 (PMC4883996; doi:10.1186/s12284-014-0022-5)
Supplement: Supplementary file 5 — Additional file 5:Table S4.: Gene distribution of Tongil. (DOCX 20 KB) [file 12284_2014_22_MOESM5_ESM.docx]

Table S4 Gene distribution of Tongil

| Chromosome | *Indica* | |  | *Japonica* | |  | Unknown | | Total |
| --- | --- | --- | --- | --- | --- | --- | --- | --- | --- |
|  | No. of genes | % |  | No. of genes | % |  | No. of genes | % |  |
| 1 | 3,614 | 77.9 |  | 953 | 20.5 |  | 75 | 1.6 | 4,642 |
| 2 | 2,553 | 68.4 |  | 1,178 | 31.6 |  | 2 | 0.1 | 3,733 |
| 3 | 3,475 | 85.7 |  | 548 | 13.5 |  | 31 | 0.8 | 4,054 |
| 4 | 2,713 | 93.2 |  | 197 | 6.8 |  | - | 0.0 | 2,910 |
| 5 | 2,440 | 90.9 |  | 245 | 9.1 |  | - | 0.0 | 2,685 |
| 6 | 2,659 | 97.2 |  | 77 | 2.8 |  | 1 | 0.0 | 2,737 |
| 7 | 2,485 | 96.4 |  | 94 | 3.6 |  | - | 0.0 | 2,579 |
| 8 | 2,261 | 99.9 |  | - | 0.0 |  | 2 | 0.1 | 2,263 |
| 9 | 1,858 | 99.3 |  | 14 | 0.7 |  | - | 0.0 | 1,872 |
| 10 | 1,390 | 77.0 |  | 416 | 23.0 |  | - | 0.0 | 1,806 |
| 11 | 2,012 | 97.0 |  | 62 | 3.0 |  | 1 | 0.0 | 2,075 |
| 12 | 1,921 | 100.0 |  | - | 0.0 |  | - | 0.0 | 1,921 |
| Total or Ave. | 29,381 | 88.3 |  | 3,784 | 11.4 |  | 112 | 0.3 | 33,277 |
