# Supplementary material for: Defining the genome structure of `Tongil' rice, an important cultivar in the Korean "Green Revolution"
Source: Rice (N Y). 2014 Sep 14;7:22. doi: 10.1186/s12284-014-0022-5 (PMC4883996; doi:10.1186/s12284-014-0022-5)
Supplement: Supplementary file 6 — Additional file 6: Figure S2.: GO analysis according to the cellular components category of Tongil genes corresponding to indica/japonica sequences. (DOCX 22 KB) [file 12284_2014_22_MOESM6_ESM.docx]

Figure S2 GO analysis according to the ‘cellular components’ category of Tongil genes corresponding to *indica*/*japonica* sequences
