# Supplementary material for: Defining the genome structure of `Tongil' rice, an important cultivar in the Korean "Green Revolution"
Source: Rice (N Y). 2014 Sep 14;7:22. doi: 10.1186/s12284-014-0022-5 (PMC4883996; doi:10.1186/s12284-014-0022-5)
Supplement: Supplementary file 9 — Additional file 9: Figure S5.: Copy number of SSR motif families in Tongil. (DOCX 1 MB) [file 12284_2014_22_MOESM9_ESM.docx]

Figure S5 Copy number of SSR motif families in Tongil

| *Indica*  *(%)* | 4048 (92.6) | 3440 (92.6) | 1253 (89.2) | 1053 (89.2) | 963 (88.9) | 919 (88.8) | 848 (88.5) | 815 (87.1) | 794 (89.4) | 773 (90.1) | 666 (88.8) | 517 (88.7) | 352 (89.3) | 335 (91.8) | 329 (91.4) | 290 (88.4) | 282 (85.7) | 261 (87.3) | 249 (89.9) | 191 (95.5) |
| --- | --- | --- | --- | --- | --- | --- | --- | --- | --- | --- | --- | --- | --- | --- | --- | --- | --- | --- | --- | --- |
| *Japonica*  *(%)* | 312 (7.1) | 267 (7.2) | 151 (10.8) | 125 (10.6) | 110 (10.2) | 111 (10.7) | 105 (11.0) | 117 (12.5) | 90 (10.1) | 80 (9.3) | 83 (11.1) | 65 (11.1) | 42 (10.7) | 29 (7.9) | 30 (8.3) | 38 (11.6) | 42 (12.8) | 35 (11.7) | 26 (9.4) | 9 (4.5) |
| Unknown  (%) | 13  (0.3) | 8  (0.2) | 0  (0.0) | 2  (0.2) | 10  (0.9) | 5  (0.5) | 5  (0.5) | 4  (0.4) | 4  (0.5) | 5  (0.6) | 1  (0.1) | 1  (0.2) | 0  (0.0) | 1  (0.3) | 1  (0.3) | 0  (0.0) | 5  (1.5) | 3  (1.0) | 2  (0.7) | 0  (0.0) |
