# Supplementary material for: Defining the genome structure of `Tongil' rice, an important cultivar in the Korean "Green Revolution"
Source: Rice (N Y). 2014 Sep 14;7:22. doi: 10.1186/s12284-014-0022-5 (PMC4883996; doi:10.1186/s12284-014-0022-5)
Supplement: Supplementary file 11 — Additional file 11: Table S5.: Comparison of alleles of yield-related genes in Tongil and its parents. (DOCX 20 KB) [file 12284_2014_22_MOESM11_ESM.docx]

Table S5 Comparison of alleles of yield-related genes in Tongil and its parents

| Gene | Gene name | Tongil | Yukara | TN1 | IR8 |
| --- | --- | --- | --- | --- | --- |
| *qSW5* | QTL for Seed width on chromosome 5 | **I**^a^ | **J** | **I**^a^ | **I**^a^ |
|  |  |  |  |  |  |
| *Gn1a* | Grain number in chromosome 1 | **I** | **J** | **I** | **I**^b^ |
|  |  |  |  |  |  |
| *Ghd7* | Grain number, plant height and heading date | **I** | **J** | **I** | **I** |
|  |  |  |  |  |  |
| *sd1* | Semi Dwarf 1 | **I** | **J** | **I** | **I** |
|  |  |  |  |  |  |
| *GS3* | Grain size 3 | **I** | **J** | **I** | **I** |
| *GW2* | Grain width and weight | **I** | **J** | **I** | **I** |
| *S5* | Hybrid Sterility 5 | **I** | **J** | **I** | **I** |

a: *indica* -II type

b: different *indica* type allele with Tongil and TN1
