# Supplementary figures and images for: Defining the genome structure of `Tongil' rice, an important cultivar in the Korean "Green Revolution"
Source: Rice (N Y). 2014 Sep 14;7:22. doi: 10.1186/s12284-014-0022-5 (PMC4883996; doi:10.1186/s12284-014-0022-5)

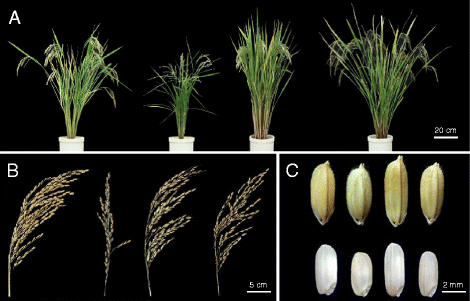

Supplement: Supplementary file 12 — Authors’ original file for figure 1 [file 12284_2014_22_MOESM12_ESM.gif]

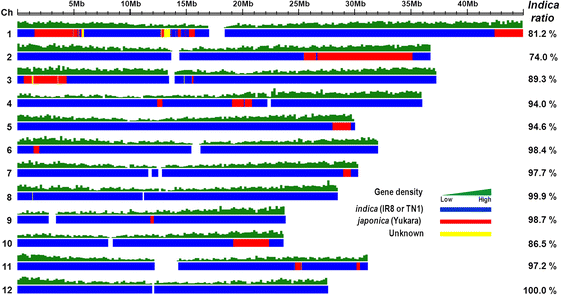

Supplement: Supplementary file 13 — Authors’ original file for figure 2 [file 12284_2014_22_MOESM13_ESM.gif]

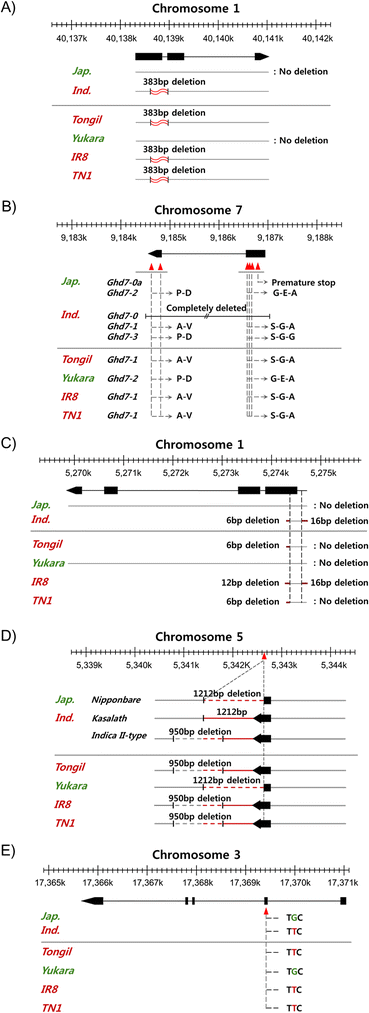

Supplement: Supplementary file 14 — Authors’ original file for figure 3 [file 12284_2014_22_MOESM14_ESM.gif]
